# Conversion of methionine biosynthesis in *Escherichia coli* from trans- to direct-sulfurylation enhances extracellular methionine levels

**DOI:** 10.1186/s12934-023-02150-x

**Published:** 2023-08-11

**Authors:** Nadya Gruzdev, Yael Hacham, Hadar Haviv, Inbar Stern, Matan Gabay, Itai Bloch, Rachel Amir, Maayan Gal, Itamar Yadid

**Affiliations:** 1 Migal - Galilee Research Institute, Kiryat Shmona, 11016 Israel; 2 Department of Oral Biology, Goldschleger School of Dental Medicine, Faculty of Medicine, Tel Aviv University, Tel Aviv, 6997801 Israel; 3 Tel-Hai College, Upper Galilee, 1220800 Israel

**Keywords:** Methionine biosynthesis, Trans-sulfurylation, Direct-sulfurylation, Metabolic engineering, *E. coli*

## Abstract

Methionine is an essential amino acid in mammals and a precursor for vital metabolites required for the survival of all organisms. Consequently, its inclusion is required in diverse applications, such as food, feed, and pharmaceuticals. Although amino acids and other metabolites are commonly produced through microbial fermentation, high-yield biosynthesis of L-methionine remains a significant challenge due to the strict cellular regulation of the biosynthesis pathway. As a result, methionine is produced primarily synthetically, resulting in a racemic mixture of D,L-methionine. This study explores methionine bio-production in *E. coli* by replacing its native trans-sulfurylation pathway with the more common direct-sulfurylation pathway used by other bacteria. To this end, we generated a methionine auxotroph *E. coli* strain (MG1655) by simultaneously deleting *metA* and *metB* genes and complementing them with *metX* and *metY* from different bacteria. Complementation of the genetically modified *E. coli* with *metX/metY* from *Cyclobacterium marinum* or *Deinococcus geothermalis*, together with the deletion of the global repressor *metJ* and overexpression of the transporter *yjeH*, resulted in a substantial increase of up to 126 and 160-fold methionine relative to the wild-type strain, respectively, and accumulation of up to 700 mg/L using minimal MOPS medium and 2 ml culture. Our findings provide a method to study methionine biosynthesis and a chassis for enhancing L-methionine production by fermentation.

**Supplementary Information:**

The online version contains supplementary material available at 10.1186/s12934-023-02150-x.

## Introduction

Methionine is a sulfur-containing amino acid that plants, fungi and bacteria synthesize, but not vertebrates; thus, it is considered an essential amino acid. Although it is one of the less abundant amino acids in proteins [1], its hydrophobic nature contributes significantly to the stabilization of proteins’ structure [2, 3]. Methionine also plays an essential role in initiating mRNA translation and indirectly regulates various cellular processes by serving as the precursor of *S*-adenosyl-methionine (SAM), a biological methyl group donor [4, 5]. As an essential amino acid for vertebrates, addition of L-methionine for food, feed or other biotechnological applications is often necessary since balanced amounts of methionine must be consumed [6, 7]. However, microbial production of methionine beyond physiological levels is challenging due to its high cellular energy demands and the strict cellular regulation of its synthesis and accumulation [8–10]. The primary method to produce methionine for food and feed supplementation involves chemical synthesis, resulting in a racemic mixture of D,L-methionine and additional toxic compounds that must be removed from the final product. Thus, there is increasing demand to produce the natural form of L-methionine through an efficient bio-fermentation process [11, 12].

### Enhancement of methionine production in ***E. coli***

**Fig. 1:**
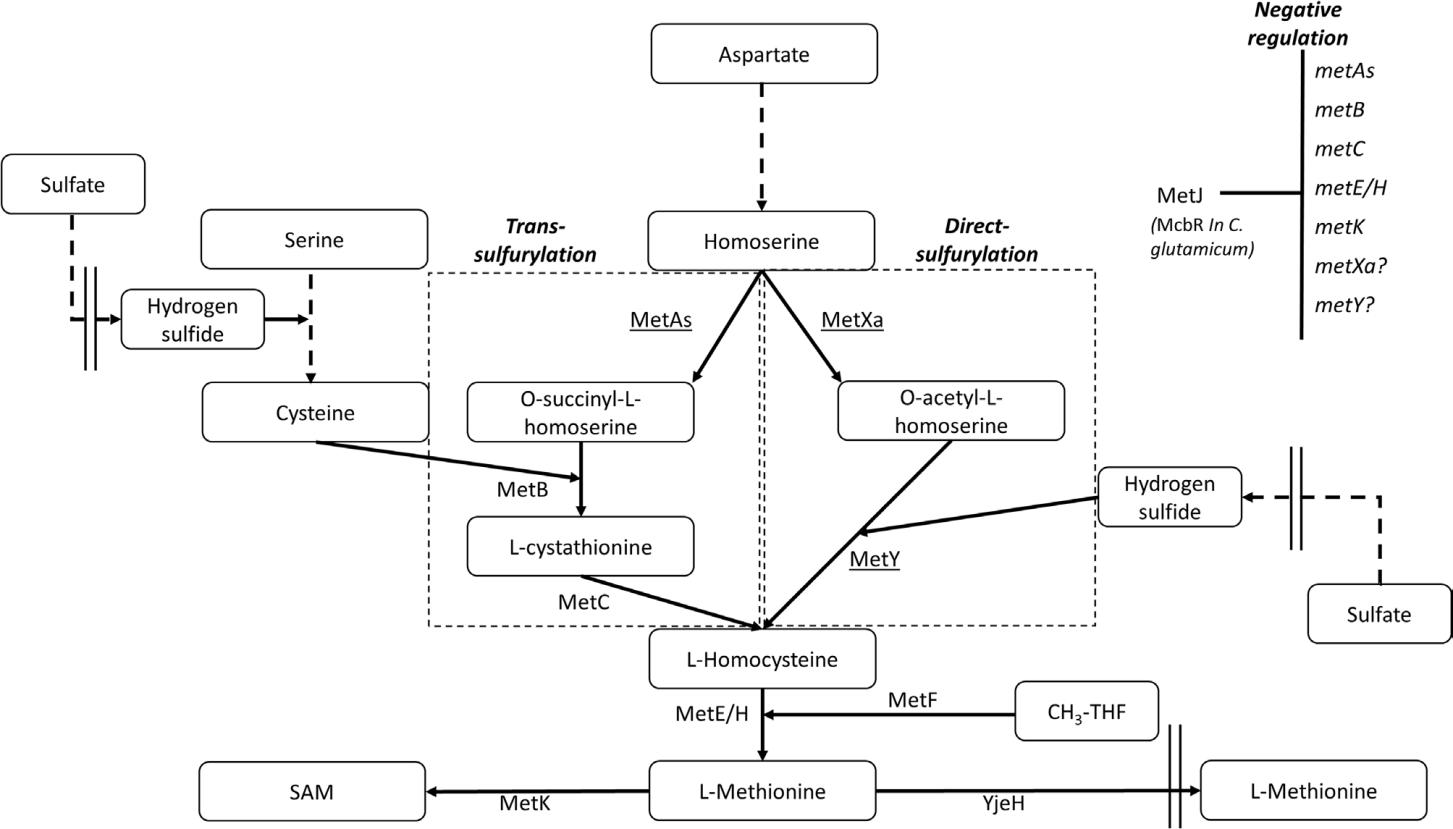
Direct- and trans-sulfurylation of methionine biosynthesis in bacteria. The first step in methionine biosynthesis involves the activation of homoserine through an acylation step. Two enzymes encoded by *metA*s and *metX*a genes [31] activate homoserine. The enzyme homoserine succinyl transferase (HST, MetAs) converts homoserine and succinyl-CoA into *O*-succinyl-L-homoserine (OSH). The enzyme homoserine acetyl transferase (HAT, MetXa) converts homoserine and acetyl-CoA into *O*-acetyl-L-homoserine (OAH). In the trans-sulfurylation pathway, cysteine and *O*-succinyl-L-homoserine (OSH) are converted into cystathionine by cystathionine-γ-synthase (CgS, MetB). Cystathionine is converted into homocysteine by cystathionine-β-lyase CbL (MetC). In the direct-sulfurylation pathway, OAH is converted into homocysteine by O-acetylhomoserine sulfhydrylase (OAHS, MetY). Metabolites in the pathways are boxed. MetJ and McbR are master negative regulators in *E. coli* and *C. glutamicum*, respectively. Additional abbreviations: MetF − 5,10-methylenetetrahydrofolate reductase, MetK - S-adenosylmethionine synthase, MetE - Cobalamin-independent methionine synthase, MetH - Methionine synthase, YjeH - L-methionine exporter, SAM - *S*-adenosyl-methionine

Efforts to produce methionine in bacteria have mainly focused on the clearance of negative regulation, controlling the metabolic flux in and out of the pathway and removing feedback inhibition of enzymes that comprise part of the biosynthesis pathway [9, 13–16]. For instance, disruption of *metJ*, which is a master regulator of the methionine pathway, together with overexpression of the genes encoding for MetA and the methionine-exporter YjeH (Fig. 1), resulted in an approximately ten-fold improvement in the production of L-methionine in *E. coli* [17, 18]. Additionally, enhancing the synthesis of upstream precursors required for methionine biosynthesis, alongside the simultaneous alteration of multiple pathways, was shown to be important for an optimized methionine bioproduction [8, 19, 20]. Moreover, modifications of regulatory elements, controlling the expression of multiple related genes, and supplementation of the bacterial growth medium with specific metabolites that were identified as limiting factors all resulted in a substantial increase of methionine levels of up to 18 g/L [9, 21–23].

### Bacterial direct- and ***trans***-sulfurylation pathways for biosynthesis of methionine

Trans- and direct-sulfurylation are the two main pathways for sulfur assimilation in bacterial methionine biosynthesis. As the names imply, the two pathways differ in the sulfur assimilation steps [24–28]. In trans-sulfurylation, homoserine is converted to L-homocysteine in three steps that are catalyzed by the enzymes MetA, MetB, and MetC, which are also known as L-homoserine *O*-succinyl transferase (HST; EC 2.3.1.46), cystathionine gamma synthase (CgS; EC 2.5.1.48), and cystathionine beta lyase (CbL; EC 4.4.1.13), respectively (Fig. 1). MetA synthesizes *O*-succinyl or *O*-acetyl L-homoserine, and MetB uses cysteine and *O*-succinyl L-homoserine to form cystathionine. In this pathway, inorganic sulfur in the form of hydrogen sulfide is first incorporated into cysteine by the enzyme *O*-acetylserine sulfhydrylase A (CysK), such that cysteine serves as the sulfur donor for the following synthesis of methionine [29]. MetC converts cystathionine into L-homocysteine (Fig. 1).

In the direct-sulfurylation pathway, L-homoserine is converted into L-homocysteine in only two steps, catalyzed by the enzymes MetX and MetY, known as L-homoserine *O*-acetyltransferases (HAT; EC 2.3.1.31) and *O*-acetylhomoserine sulfhydrylase (OAHS; EC 2.5.1.49), respectively. MetX produces *O*-acetyl L-homoserine from homoserine and acetyl-CoA, while MetY combines *O*-acetyl L-homoserine with an inorganic sulfur in the form of hydrogen sulfide to form L-homocysteine; thus, the latter does not rely on cysteine as the sulfur source (Fig. 1). Similar to sulfur assimilation during cysteine biosynthesis, *E. coli* can reduce sulfate to sulfide and process sulfur from various other sources such as sulfite, sulfide, or thiosulfate [30].

### Exploring alternative pathways for sulfur assimilation in ***E. coli***

While most bacteria use the direct-sulfurylation pathway for methionine biosynthesis, *E. coli* utilizes the trans-sulfurylation pathway [24, 28, 32]. This pathway is less parsimonious in terms of the number of steps and proteins involved and depends on sulfur to be first assimilated into cysteine [24, 33, 34]. To control the methionine biosynthesis pathway in *E. coli*, key enzymes in the trans-sulfurylation pathway are strictly regulated and feedback-inhibited by methionine and SAM [24, 35]. Thus, while *E. coli* is a valuable workhorse in synthetic biology, the trans-sulfuration pathway might be a limiting step for using it to bio-produce methionine. An alternative approach to bypass the inherent regulation of *E. coli* on methionine biosynthesis involves introducing genes from various organisms that are less prone to inhibition. Indeed, it was shown that MetX from *Leptospira meyeri* is not feedback-inhibited by methionine or SAM [36]. Previous studies have demonstrated that the introduction of genes involved in the direct-sulfurylation pathway can significantly enhance methionine production in *E. coli* [37]. Moreover, it has been observed that genes sourced from bacteria utilizing direct-sulfurylation can serve to complement for methionine auxotrophy in *E. coli* [38, 39].

Therefore, the primary objective of this study was to investigate the impact on methionine biosynthesis of concurrently deleting both *metA* and *metB* genes and replacing them with *metX* and *metY*. This genetic modification would facilitate a complete transition of *E. coli* from trans- to direct-sulfurylation, thereby affecting methionine levels. Our findings demonstrate that the deletion of the *metA/B* genes in *E. coli* MG1655 resulted in a methionine auxotroph that could be complemented by the insertion of met*X/Y* genes from various sources. Furthermore, we found that the origin of the genes and their catalytic activity were closely associated with the ability of *E. coli* to produce methionine, leading to a significant increase in intra- and extra-cellular methionine levels.

## Results

### Engineering of methionine auxotroph ***E. coli***

**Fig. 2:**
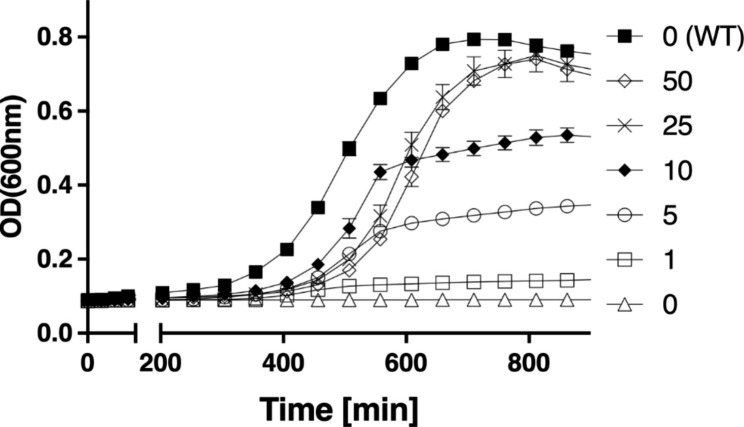
*E. coli*Δ*metAB* is auxotrophic for methionine. WT and Δ*metAB* were grown in a minimal MOPS medium with or without supplementation of external methionine for 900 min at 37 °C. The legend shows supplemented methionine concentration in μg/ml

To explore the option of converting the *E. coli* methionine biosynthesis pathway from trans- to direct-sulfurylation, we deleted two essential genes in the methionine pathway of *E. coli*, *met*A and *metB*, encoding for the enzymes HST and CGS, respectively (Fig. 1). This deletion generated a methionine auxotroph *E. coli* strain (Δ*metAB*). Figure 2 shows growth curves of Δ*metAB* in a MOPS minimal medium containing glucose and ammonium chloride as the carbon and nitrogen sources, respectively, and K_2_SO_4_ as the main sulfur source. To test the effect of methionine on the growth rate, we supplemented varying concentrations of external methionine, as indicated in Fig. 2. While the WT bacteria grew normally without supplementation of methionine, the Δ*metAB* methionine auxotroph was unable to grow. However, Δ*metAB* growth was rescued with the addition of methionine. At a concentration of 25 μg/ml methionine, Δ*metAB* growth reached maximal levels and the cell density resembled that of the WT bacteria, demonstrating that methionine was indeed the limiting growth factor.

### Complementation of ***E. coli*** Δ***met***AB by ***metX*** and ***metY*** genes from different bacterial genomes

With the aim of exploring *E. coli’s* ability to synthesize methionine via the direct-sulfurylation pathway using the Met*X* and Met*Y* enzymes, we cloned four different *metY/X* gene pairs to complement the Δ*metAB* methionine auxotroph bacteria. The selection of *metY/X* gene pairs was based on a previously characterized dataset of MetX enzymes from various bacteria [31]. We applied two criteria to select the strains. First, the MetX enzymes should exhibit a range of catalytic activity between 10^3^ and 10^4^ nmol·min^− 1^·mg^− 1^ (of *O*-acetyl L-homoserine formation). Second, the relevant bacterial genome should contain a sequence for the counterpart *metY* gene adjacent to the *metX* gene, indicating a mini operon of two genes with coordinated expression and function.

Based on the above rationale, we selected *metY*/*metX* pairs from four bacterial strains: (i) *Corynebacterium glutamicum* (CG), (ii) *Leptospira interrogans* (LI), (iii) *Cyclobacterium marinum* (CM) and (iv) *Deinococcus geothermalis* (DG). Our hypothesis was that higher activity of MetX could result in increased methionine production. To this end, we incorporated MetX and MetY from CM and DG which exhibit higher activity and from LI and CG, which shows lower activity [31]. The genes from LI and CG have been previously characterized and demonstrated to be expressed in *E. coli* [37, 38]. Table 1 summarizes the UniProt entry identifiers of each protein. Sequence identity between the various MetX and MetY proteins is provided in Table S1 of the supplementary information.

**Table 1:** UniProt identifiers of the enzymes encoded by the genes used to complement the methionine auxotroph bacteria

Bacterial strain	*metX* UniProt ID	*metY* UniProt ID
*Leptospira interrogans*	Q8F4I0	P94890
*Corynebacterium glutamicum*	O68640	Q79VI4
*Deinococcus geothermalis*	Q1J115	Q1J114
*Cyclobacterium marinum*	G0J5N4	G0J5N3

**Fig. 3:**
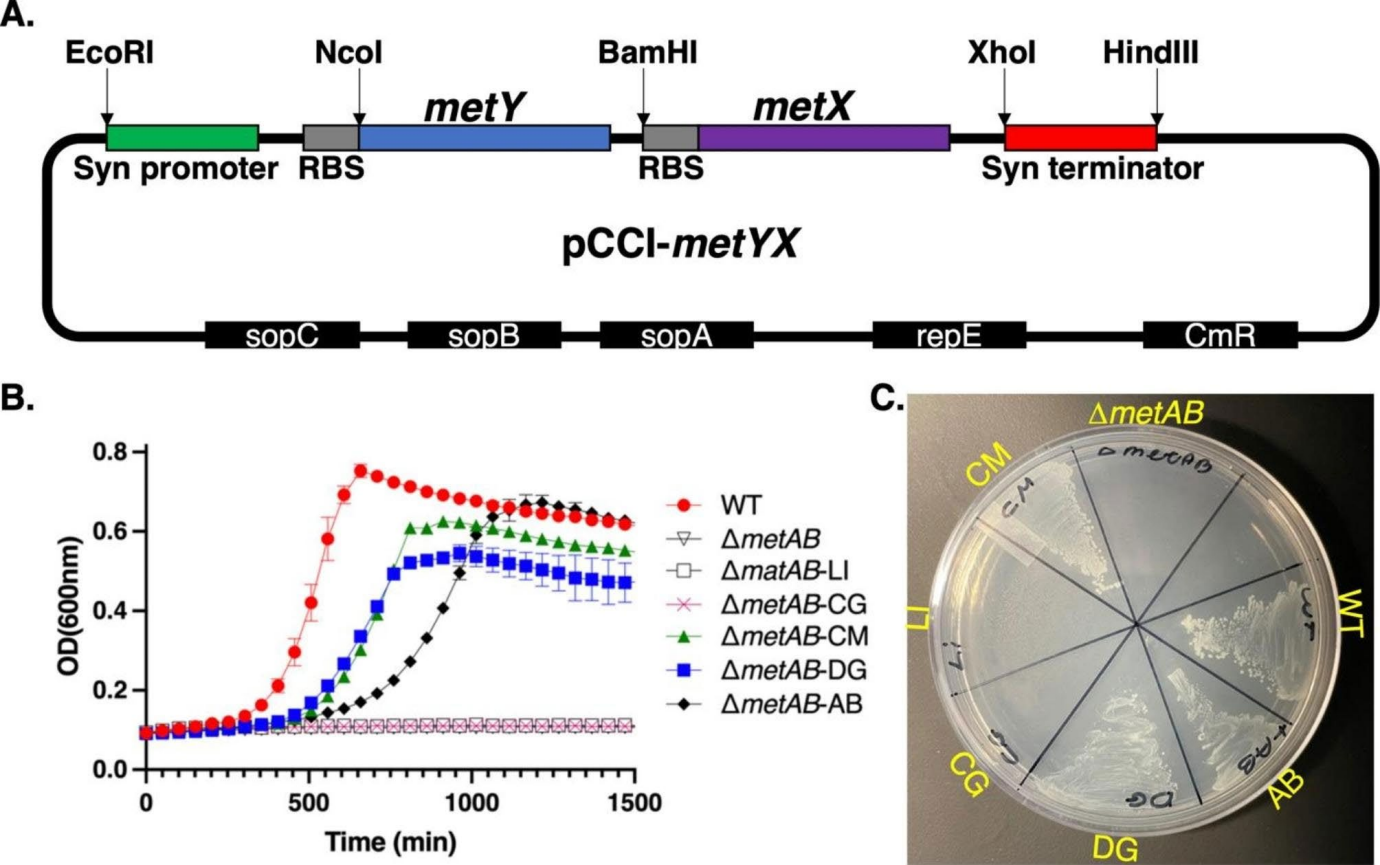
Complementation of Δ*metAB* with *metX/Y* gene pairs. (**A**) Schematic illustration of the synthetic *metYX* operon on a low-copy plasmid used to complement Δ*metAB*. A synthetic operon consisting of the *metY* and *metX* genes was constructed by adding a synthetic constitutive promoter, a ribosome binding site (RBS) for each gene, and a synthetic terminator. Restriction sites were included to facilitate rearrangement and analysis of mutant genes. (**B**) Growth curves of the complemented Δ*metAB* strains on a minimal MOPS medium. WT: *E. coli* MG1655; Δ*metAB*: WT with deletion of the *metAB* genes; Δ*metAB*-DG/CM/LI/CG: Δ*metAB* complemented with a pCCl plasmid expressing *metX* and *metY* of the indicated bacterial strain. Δ*metAB-*AB: WT with deletion of the *metAB* genes complemented with a pCCl plasmid expressing *E. coli’s metA* and *metB*. (**C.**) Growth of the complemented Δ*metAB* strains on a MOPS minimal-medium agar plate incubated at 37 °C for 24 h

To design a uniform construct for expression in *E. coli*, the *metX* and *metY* genes were codon-optimized and cloned into a pCCl plasmid [40, 41] downstream to a synthetic constitutive promoter, a synthetic ribosome binding site (RBS) and followed by a synthetic terminator. At this stage, we maintained the gene arrangement as observed in the four different genomes, where *metX* is consistently positioned after *metY*. The synthetic promoter ensured constitutive expression, and the use of the pCCI plasmid enabled the maintenance of the inserted genes at low to a single-copy number, similar to the genomic copy number of the corresponding genes. To facilitate a comparison and ensure similar regulation, we also constructed a similar plasmid harboring the wild-type *metAB* genes of *E. coli*. This allowed us to assess complementation from the same genetic construct under comparable regulatory conditions, enabling a reliable and informative analysis. Figure 3A shows a schematic illustration of the constructed operon. The complete gene sequences and their accession numbers are depicted in the supplementary information. Following the transformation of the auxotroph bacteria with the plasmids, we evaluated the ability of the complemented Δ*metAB* strain to grow in a liquid minimal MOPS medium. As shown in Fig. 3B, the methionine auxotroph *ΔmetAB* bacteria was successfully complemented with *metY/X* from DG and CM (*ΔmetAB*-DG and *ΔmetAB*-CM, respectively) and reached a similar growth rate and final cell density as those of the WT after ~ 800 min. The complemented bacteria carrying *metY*/*X* of LI and CG (Δ*metAB*-LI and Δ*metAB*-CG in Fig. 3B) did not grow under these conditions. Moreover, the plasmid carrying the wild-type metA/B ((*ΔmetAB*-AB) shows somewhat slower growth. Similar growth patterns were observed on minimal medium agar plates (Fig. 3C), indicating that the CM- and DG-complemented strains were able to produce methionine at levels sufficient to maintain their growth.

**Fig. 4:**
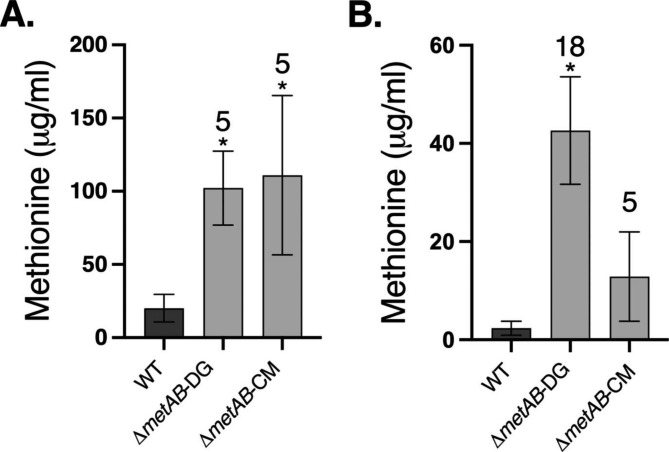
Biosynthesis of methionine by *E. coli* Δ*metAB* complemented with *metY/X* pairs. WT and complemented *E. coli* Δ*metAB* were grown in a minimal MOPS medium at 37 °C for 24 h, after which the cells were separated from the growth medium. The amount of methionine in each fraction was evaluated using GC-MS. (**A**) Intracellular methionine accumulated by WT *E. coli*, Δ*metAB*-DG and Δ*metAB*-CM, reported as μg/ml. (**B**) Extra-cellular methionine accumulated in the growth media by WT *E. coli*, Δ*metAB*-DG and Δ*metAB*-CM, reported as μg/ml. Peak areas were normalized to ribitol internal control, and total methionine levels were calculated according to the standard methionine calibration curves. The results are presented as means ± SD of three to four replicates for each sample. Significance between WT and the different bacterial strain was calculated according to the Student’s t-test (P < 0.05) and is identified by an asterisk. The numbers on top of the bars indicate the fold increase relative to the WT in each panel

To quantify intracellular and extracellular methionine levels in the complemented bacteria, the methionine levels were evaluated using GC-MS and compared to those of the WT bacteria. The Δ*metAB*-DG *and ΔmetAB*-CM strains exhibited a five-fold enhancement of intracellular methionine levels compared to WT (Fig. 4A). Analysis of the extracellular methionine in the growth medium indicated that Δ*metAB*-DG exhibited significantly enhanced accumulation of extracellular methionine as compared to WT (18 fold). Although the difference in methionine accumulation was not significant in the *ΔmetAB*-CM strain compared to the control WT bacteria, its average obtained from four repeats also showed a five-fold increase (Fig. 4B).

### Evaluation of intra- and extracellular methionine levels of ***E. coli*** with a deletion of ***metJ*** and overexpression of ***yjeH***

After establishing *E. coli* strains expressing the *metY*/*X* of DG and CM in a Δ*metAB* background, we further explored the effect of additional genetic variations related to the methionine biosynthetic pathway. More specifically, we deleted the gene encoding for the MetJ repressor that is known to strictly repress the transcription of multiple genes in the *E. coli* methionine biosynthetic pathway (*metA*, *metB*, *metC*, *metE/H*, Fig. 1) in response to elevated methionine levels [42, 43].

**Fig. 5:**
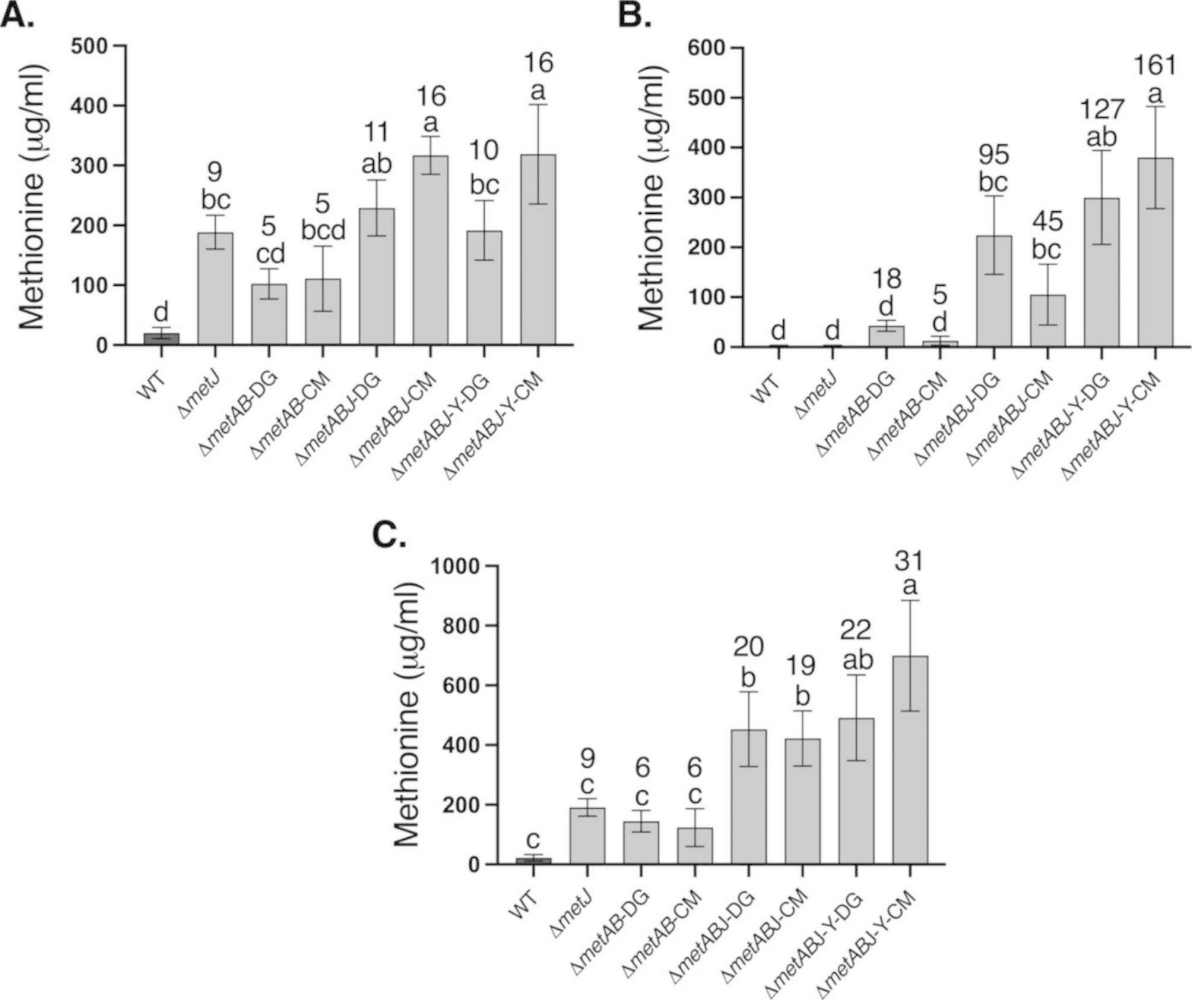
Production of methionine by Δ*metABJ* overexpressing YjeH and complemented by *metY/X* from CM or DG. Comparison of: (**A**) intracellular; (**B**) extracellular; and (**C**) total methionine levels that were quantified by GC-MS. Peak areas were normalized to ribitol internal control, and total methionine levels were calculated according to the standard methionine calibration curves. The results are presented as means ± SD of three or four replicates for each sample. Significance between bacterial strains was calculated according to the Tukey’s HSD test (p < 0.05) and is identified by different small letters. The numbers on top of the bars in each panel indicate the fold-increase relative to the WT in each panel

To evaluate the ability of the engineered strains to produce methionine, the bacteria were cultured in a minimal medium until reaching OD_600_ = 2.5. The intracellular and extracellular levels of methionine were evaluated using GC-MS (Fig. 5). Deletion of *metJ* on a WT background led to a 9-fold enhancement in the level of intracellular methionine relative to WT (Fig. 5A), while no change was detected in the extracellular methionine levels (Fig. 5B). Deletion of *metJ* on the Δ*metAB*-CM background led to a 16- and 45-fold increase in intracellular and extracellular methionine levels, respectively, while in Δ*metABJ*-DG, the levels increased by 11- and 95-fold, respectively, relative to WT (Fig. 5A-B).

To further increase methionine levels in the growth medium and reduce the level of inhibition on methionine-feedback sensitive enzymes or regulators, we also targeted the *E. coli* methionine exporter protein YjeH. This exporter was shown to have a strong positive effect on extracellular methionine accumulation and to reduce the methionine content inside the bacterial cells [18]. Therefore, its gene was cloned to facilitate overexpression and to enable enhanced methionine efflux to the medium. The *metY/X* plasmid carrying *metY/X* from DG or CM was than introduced into Δ*metABJ*-Y, resulting in Δ*metABJ*-Y-CM and Δ*metABJ*-Y-DG. This procedure led to similar intracellular methionine levels as in Δ*metAB*-CM and Δ*metABJ*-DG, but it significantly increased the levels of extracellular methionine by 161- and 127-fold, respectively, relative to WT (Fig. 5B). Overall, methionine levels (combining the intra and extracellular methionine) increased by up to 31-fold over the WT and reached up to 700 μg/ml (Fig. 5C).

### Bioavailability of extracellular methionine secreted from the engineered Δ***metABJ-Y***-DG and Δ***metABJ-Y*** -CM strains and its potential use as a methionine supplement

**Fig. 6:**
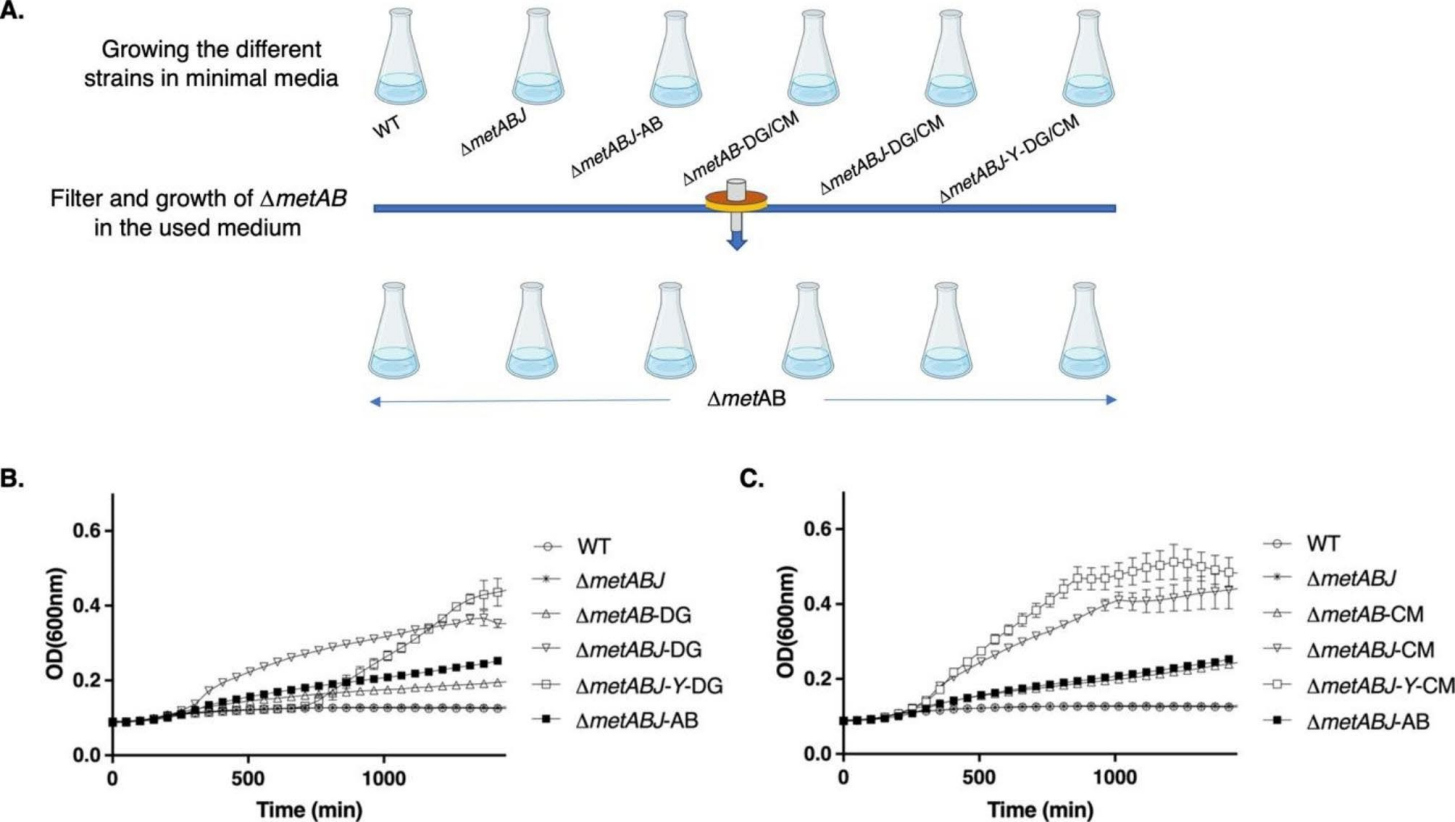
Growth curves of methionine-auxotroph*E. coli* in spent medium of each strain. (**A**) Illustration of the experimental scheme used to evaluate methionine level in the medium following the growth of each strain. (**B**) Growth curves in medium from DG strains. (**C**) Growth curves in medium from CM strains. All curves show the growth of the auxotroph *E. coli* Δ*metAB* in fresh MOPS minimal medium supplemented with spent and filtered medium following the growth of the indicated strains

To confirm the bioavailability of the extracellular methionine secreted from each engineered strain using an orthogonal approach, we collected the spent medium at the end of the bacterial growth phase of each strain and filtered it through a 0.22 μm membrane. The filtered spent medium was then added to a fresh methionine-free minimal MOPS medium at a 1:1 ratio. Thus, methionine could only be delivered from the filtered spent medium (Fig. 6A). The mixed medium was then evaluated for its ability to support the growth of *ΔmetAB* auxotroph. Figure 6 shows the growth curves of the Δ*metAB* auxotroph bacteria grown in the mixed medium originated from the spent medium of the DG- (Fig. 6B) and CM- (Fig. 6C) complemented strains and compered to the mixed medium originated from the spent medium of Δ*metAB*-WT. The highest cell density was observed when the *ΔmetAB* auxotroph was cultured with spent medium originating from *ΔmetABJ*-Y-CM/DG, suggesting that this strain exported the highest methionine levels. On the other hand, the *ΔmetAB* auxotroph did not grow with medium originated from the WT, indicating for the lack of methionine in its medium. Both results are congruent with the methionine levels that were measured for these strains using GC-MS (Fig. 5).

## Discussion

Various microbial cells are utilized to produce amino acids [44]. Among them, *E. coli* has gained significant attention as a promising organism for enhancing the bioproduction of natural amino acids, including L-methionine [45–49]. Despite numerous studies and significant advances in exploiting the potential of *E. coli* in this regard, the efficient production of L-methionine through bacterial fermentation remains a challenge. Previous studies focused primarily on metabolic engineering of *E. coli* W3110, achieved significant methionine levels of up to 18 g/L by utilizing medium supplemented with yeast extract and vitamins [22, 48]. In our present study, we relied on MOPS minimal medium and utilized *E. coli* MG1655 [9, 17], a closely related strain that has demonstrated its potential as a robust producer of bioproducts and has been interchangeably used with *E. coli* W3110 in various studies [50–52].

Regardless of the bacterial strain, efforts to enhance methionine levels in *E. coli* mostly involve the engineering of multifaceted cellular pathways that aim to release negative feedback regulation alongside optimizing the utilization of methionine precursors. While this strategy has resulted in significant improvements, it relies on harnessing the natural trans-sulfurylation pathway of *E. coli*. Methionine biosynthesis by the direct-sulfurylation pathway is much more abundant in the bacterial kingdom. However, it has been characterized in a relatively limited number of strains [24]. Moreover, only limited data is available on the catalytic properties of the central MetY enzymes [33, 53, 54] and their related 3D structures [28, 55–57].

Enzymes of the direct-sulfurylation pathways are versatile and can process various substrates [25, 26]. Indeed, previous studies showed that *E. coli* can grow with such enzymes [36, 38]. Thus, the current study aimed to replace the natural enzymes in *E. coli* with their counterpart from the direct-sulfurylation pathway. To that end, we explored the ability to complement the methionine auxotroph *ΔmetAB* strain with *metX* and *metY* enzymes from various bacteria completely forming direct-sulfurylation within the *E. coli*. The heart of the effort involved replacing the enzymes HST and CgS with HAT and OAHS. We inserted the genes encoding MetX (HAT) and MetY (OAHS) into the methionine auxotroph strain via a plasmid containing a synthetic mini operon of *metY* followed by *metX* (Fig. 3A). The transformation of the auxotrophic strain with a plasmid carrying the *metY/X* genes from DG and CM allowed for bacterial growth without the external addition of methionine. On contrary, the insertion of *metX* and *metY* from CG and LI failed to complement the methionine auxotroph *E. coli*. Of note, these enzymes were previously reported to complement D*metA* methionine auxotroph bacteria with *metXY* from CG [38] or a D*metAB* methionine auxotroph bacteria, with *metXY* from LI, however, in this particular case, the complementation occurred with a slow generation time [39]. This difference could be due to these enzymes’ reduced efficiency compared to the enzymes of DG and CM. However, it is possible that the activity of these enzymes requires additional co-factors and/or certain conditions that are not present in the context of the trans-sulfurylation pathway within *E. coli*. An additional explanation could be that a decreased expression level, misfolding leading to protein aggregation or faster degradation, contributed to the inability of the bacteria to grow. Regardless of the exact mechanism, this finding suggests that large variability exists in the activity of the different enzymes when complemented into *E. coli*. Therefore, the screening of additional genes from multiple organisms may further benefit methionine accumulation.

Several mechanisms could explain the higher methionine accumulation in the strains complemented with enzymes from the DG and CM strains relative to the WT containing the *metA* and *metB* genes on the same construct (Fig. 6B-C). *E. coli* employs stringent regulation mechanisms to tightly control methionine production. This regulation takes place at the DNA level, involving specific transcription factors like *metJ*, and through the inhibition of protein activity by methionine or related metabolites such as SAM. In our study, we introduced MetX and MetY genes under the control of a constitutive promoter that remains unaffected by changes in methionine concentration. Furthermore, it is possible that the MetX and MetY enzymes display reduced sensitivity to inhibition, as suggested by Bourhy et al. [36]. However, it is worth noting that other genes in the methionine pathway may still be susceptible to inhibition by methionine and related metabolites. Consequently, this limitation could contribute to a modest increase in intracellular methionine concentrations [14, 17]. Our results indicate that accumulation of methionine beyond a certain threshold, leads to methionine export outside of the cell (Fig. 4). Indeed, deletion of *metJ* resulted in higher methionine biosynthesis in the WT strain, showing that the release of regulation at the transcription level is an important factor for enhancing methionine biosynthesis [9, 14]. Without the transcriptional regulation in the Δ*metJ* strain, deletion of *metA* and *metB* together with complementation with *metX* and *metY* (Δ*metABJ*-DG/CM) further pushed the levels of methionine above those found in Δ*metAB*-DG/CM. This finding could be due to the higher rate of enzymatic activity of *metX* in comparison to the rate-limiting enzyme *metA* in the trans-sulfurylation pathway [31] in addition to the reduced regulation of other important genes in the pathway, such as *metE* and *metH* (Fig. 1). Indeed, it was previously shown that MetX from LI expressed in *E. coli* was not affected by feedback inhibition imparted by high levels of methionine or SAM [36]. Of note, the higher levels of methionine observed in the Δ*metABJ*-Y-CM compared to Δ*metABJ*-CM suggest that excess of methionine inside the cells is controlled by other factors, some of which are yet unknown. Indeed, when the YjeH transporter was overexpressed, it enhanced the cells efflux and enabled the bacteria to produce more methionine.

The enhancement of methionine biosynthesis in the engineered *E. coli* warrants screening of additional MetX and MetY enzymes of other strains, to further characterize their ability to support methionine production. In addition, it may be possible to boost methionine levels by optimizing growth conditions with alternative sulfur sources and introducing additional modifications to the direct-sulfurylation pathway that aim to enhance metabolic flux and methionine export. Discovery of additional factors and their subsequent genetic alteration may further increase levels of methionine. These alterations can be achieved by classical strain improvement, using inhibitors such as norleucine, or by building new genetic circuits to control the expression of relevant genes. Our results show that the MetAB enzymes could be a limiting step in methionine biosynthesis regardless of additional modifications applied to the cell, and that the use of direct-sulfurylation MetYX enzymes dramatically enhanced methionine production (Fig. 5).

Additionally, our findings demonstrate that through the substitution of trans-sulfurylation with direct-sulfurylation, elevated levels of methionine can be exported and accumulated in the growth medium. This bioavailable methionine successfully supported the growth of the Δ*metAB* auxotroph bacteria and thus has promising applications in fields such as animal feed and mammalian cell culture cultivation. Notably, while the direct-sulfurylation pathway demonstrates versatility in processing sulfur sources, our study focused specifically on using potassium sulfate (K_2_SO_4_) as the main sulfur source at moderate concentrations. This exploited *E. coli’s* ability to convert it into sulfide (S^2−^). Consequently, further investigation is needed to assess the impact of alternative, non-limiting, inorganic sulfur sources such as sulfide and sulfite on growth rate and extra cellular methionine levels. Moreover, it will provide valuable insights into sulfur assimilation in the methionine biosynthesis pathway. As such, these findings pave the way for further advancements in the utilization of *E. coli* for producing L-methionine.

## Conclusions

Harnessing *E. coli* to produce L-methionine presents a promising avenue for enhanced production; however, it necessitates the modification of numerous regulatory and enzymatic bottlenecks throughout the biosynthetic pathway. Our findings suggest that by substituting the trans-sulfurylation *metA* and *metB* genes with the direct-sulfurylation *metX* and *metY* genes, methionine production in minimal medium can be significantly enhanced up to 700 mg/L.

## Materials and methods

### Bacterial strains and growth conditions

The *E. coli* strain MG1655 is referred to as the WT strain and was used in this study for all genetic manipulation. The bacteria were routinely grown in a lysogeny broth (LB) medium at 37 °C. For screening of the genetic variants, bacteria were grown in liquid or solid (supplemented with 1.5% agar) MOPS medium [58] (8.37 g/L MOPS, 0.71 g/L Tricine, 0.51 g/L NH_4_Cl, 0.05 g/L K_2_SO_4_, 2.92 g/L NaCl, 2.8 mg/L FeSO_4_, 0.074 mg/L CaCl_2_, 0.1 g/L MgCl_2_, 1 ml/L trace elements, 0.23 g/L K_2_HPO_4_, 2 g/L glucose, pH 7.3).

### Generation of methionine auxotrophic mutants

All primers used in this study are listed in the supplementary information (Table S2). Genes in MG1655 were deleted by the lambda red recombinase procedure [59], with the pKD4 plasmid carrying the Kn^R^ cassette serving as a template for PCR reactions. Mutations were verified using nearby locus-specific primers (Table S2), with the respective primers k2 or kt. Afterwards, the cassette was removed, and double/triple knockouts were further generated using a similar approach. Knockouts were generated in the following order Δ*metA*→Δ*metB*→Δ*metJ*, to form the bacterial strains Δ*met*A, Δ*met*AB and Δ*met*ABJ.

### Complementation with a plasmid carrying the *metX/Y* genes

Electrocompetent Δ*metAB*/Δ*metABJ* mutants were transformed with the pCCI plasmid carrying the *metXY* synthetic operon. Following transformation, several colonies growing on LB agar plates supplemented with 30 μg/ml chloramphenicol were tested for the presence of the correct plasmid by colony PCR, with M13F and M13R primers. Positive clones were further screened for their ability to grow in methionine-depleted minimal media (MOPS). Briefly, cultures were prepared by inoculating a 5 ml MOPS medium with a single colony grown on LB plates and incubating it overnight at 37 °C (constant orbital shaking 200 rpm). The culture was diluted 1,000-fold in a fresh MOPS medium, and 200 μl were then placed in each well of a 96-well plate (Costar). Bacteria were grown for 20 h, at 37 °C (constant orbital shaking 280 rpm), and OD_600_ was measured every 16 min, using an Infinite M200 Plate Reader (Tecan). Each sample was tested in triplicates. For control, the MOPS medium was supplemented with 5–50 μg/ml methionine (Merck). Alternatively, a single colony grown on the LB medium was spread on MOPS agar plates, and growth following 24–72 h incubation at 37 °C was visually inspected.

### Construction of a ***yjeH*** overexpression plasmid

The *yjeH* gene was amplified from genomic DNA extracted from *E. coli* MG1655 using a forward primer that adds an *Nco*I restriction site (GCGCCATGGATGAGTGGACTCAAACAAGAAC) and a reverse primer adding an *Xho*I restriction site (GCGCTCGAGTTATGTGGTTATGCCATTTTCC). The purified PCR product was digested with *Nco*I and *Xho*I and inserted into pTrcHis-a digested with the same enzymes. The correct construct was validated by sequencing.

### Qualitative evaluation of extracellular methionine levels

For qualitative analysis of the methionine concentration in the medium, WT and methionine producing mutants were grown overnight at 37 °C in a 5 ml MOPS medium and filtered through a 0.22 μm membrane to remove bacterial cells. The filtered cell-free medium was diluted two-fold in a fresh MOPS medium containing no methionine, ensuring all methionine in the new medium was secreted by the original bacteria. The new medium was tested for its ability to support the growth of a Δ*metAB* mutant in a minimal MOPS medium. Growth rate was determined by OD_600_ measurement.

### Methionine extraction from lysate and medium to evaluate intra- and extra-cellular methionine levels

To evaluate the intracellular level of methionine, amino acids were extracted from cell pellets after centrifugation of 1 ml bacterial culture, using methanol:water:chloroform at a ratio of 1:1:2.5. After centrifugation, the crude extract was separated into polar and nonpolar phases by adding 300 μl water and 300 μl chloroform and centrifuging for 10 min. A 400 μl sample from the top polar phase were vacuum-dried. To evaluate extracellular methionine levels, amino acids were extracted from 500 μl of the bacterial culture medium by adding 500 μl chloroform and centrifuging for 10 min. A 400 μl sample from the upper polar phase was vacuum-dried. The latter fraction was dissolved in 40 μl of 20 mg/ml of methoxyamine hydrochloride in pyridine and incubated at 37 °C for 2 h with vigorous shaking, followed by derivatization for 30 min in N-methyl-N(trimethylsilyl)-trifluoroacetamide at 37 °C. One μl from each sample was used for methionine-level analysis, using GC-MS.

### Evaluation of methionine levels by GC-MS

GC-MS analyses were carried out on Agilent 7890 A GC-MS coupled with a mass selective detector (Agilent 5975c), a Gerstel multipurpose sampler (MPS2), and equipped with a BP5MS capillary column (SEG; 30 m, 0.25-mm i.d., and 0.25-mm thick). For free amino acid detection, the single-ion mass method was used. Amino acid standards of 5,10, 25, 50, 100 and 200 μM were used to generate standard calibration curves, and ribitol (2 mg/ml in HPLC-grade water) was used as an internal standard. Peak areas were calculated from the standard calibration curves and normalized to the ribitol signal.

### Bacterial strains and plasmids used in this study

Table 2 shows the different engineered strains and the terminology used in this study.

**Table 2:** Bacterial strains used in the study

Name in the manuscript	Description
Wild-type, WT	*E. coli* MG1655
Δ*metAB*	*E. coli* MG1655 with a deletion of the *metA* and *metB* genes
Δ*metABJ*	*E. coli* MG1655 with a deletion of the *metA*, *metB* and *metJ* genes
Δ*metABJ-*Y	*E. coli* MG1655 with a deletion of the *metA*, *metB* and *metJ* genes overexpressing the methionine exporter YjeH
DG/CM/LI/CG	Complementation of the engineered bacteria by the pCCI plasmid expressing *metX* and *metY* of the noted bacterial strain
Δ*metAB*-AB/ Δ*metABJ-*AB	Complementation of the engineered bacteria by the pCCI plasmid expressing metA and metB from *E. coli*

### Electronic supplementary material

Below is the link to the electronic supplementary material.

**Supplementary Material 1**. **Table S1 **- Sequence identity between the four selected MetX proteins and between the four selected MetY proteins. **Table S2** - Primers used in this study.** Supplementary text 1** -? Sequences of sythetic constructs and their assigned accession number.

## Data Availability

The authors confirm that all of this study data are available within the article and its supplementary information.
